# Diffusion based multi-domain neuroimaging harmonization method with preservation of anatomical details

**DOI:** 10.1016/j.neuroimage.2025.121297

**Published:** 2025-05-26

**Authors:** Haoyu Lan, Bino A. Varghese, Nasim Sheikh-Bahaei, Farshid Sepehrband, Arthur W Toga, Jeiran Choupan, For the Alzheimer’s Disease Neuroimaging Initiative

**Affiliations:** a Laboratory of Neuro Imaging, USC Mark and Mary Stevens Neuroimaging and Informatics Institute, USC Keck School of Medicine, University of Southern California, Los Angeles, CA, USA; b Department of Radiology, USC Keck School of Medicine, University of Southern California, Los Angeles, CA, USA; c NeuroScope Inc, NY, USA

**Keywords:** Harmonization, Denoising diffusion probabilistic model, Domain adaptation, Structural MRI, Radiomics analysis, Image processing

## Abstract

In multi-center neuroimaging studies, the technical variability caused by the batch differences could hinder the ability to aggregate data across sites, and negatively impact the reliability of study-level results. Recent efforts in neuroimaging harmonization have aimed to minimize these technical gaps and reduce technical variability across batches. While Generative Adversarial Networks (GAN) has been a prominent method for addressing harmonization tasks, GAN-harmonized images suffer from artifacts or anatomical distortions. Given the advancements of denoising diffusion probabilistic model which produces high-fidelity images, we have assessed the efficacy of the diffusion model for neuroimaging harmonization. While GAN-based methods intrinsically transform imaging styles between two domains per model, we have demonstrated the diffusion model’s superior capability in harmonizing images across multiple domains with single model. Our experiments highlight that the learned domain invariant anatomical condition reinforces the model to accurately preserve the anatomical details while differentiating batch differences at each diffusion step. Our proposed method has been tested using T1-weighted MRI images from two public neuroimaging datasets of ADNI1 and ABIDE II, yielding harmonization results with consistent anatomy preservation and superior FID score compared to the GAN-based methods. We have conducted multiple analyses including extensive quantitative and qualitative evaluations against the baseline models, ablation study showcasing the benefits of the learned domain invariant conditions, and improvements in the consistency of perivascular spaces segmentation analysis and volumetric analysis through harmonization.

## 1. Introduction

Neuroimaging such as magnetic resonance imaging (MRI) data acquired from different batches, including various imaging sites or scanners, has shown promise in improving sample sizes for neuroscience studies that rely on imaging and predictive efforts (Bethlehem et al., 2022; Marek et al., 2022; Van Erp et al., 2014). However, these neuroimaging data from multiple batches are susceptible to non-biological and technical variations that occur across data acquisition from different batches, commonly known as batch effects. These batch effects can arise from disparities in acquisition protocols, scanner manufacturers, scanner drift, and hardware imperfections (Jovicich et al., 2006). The existence of batch effects can potentially pose challenges in terMS of reproducibility of neuroscience studies, generalizability of prediction algorithMS, and effective integration of radiomics-based imaging biomarkers in clinical practice (Carass et al., 2024; Grech-Sollars et al., 2015). Neglecting to consider the acknowledged interference of batch effects can result in a reduction in effectiveness, less reliable discoveries, and potentially distorted results. Various types of methods have been investigated to harmonize the neuroimaging data to remove the batch effects ranging from statistical methods focusing on correcting the feature distributions ( (Bayer et al., 2022; Fortin et al., 2016, 2017) to image processing methods focusing on harmonizing the raw MRI images directly (Cackowski et al., 2023; Chang et al., 2022; Dewey et al., 2019; Dinsdale et al., 2021; Ren et al., 2021; Roca et al., 2023a; Yao et al., 2022; Zuo et al., 2021). There are limitations commonly recognized in the harmonization tasks, such as, possibly introduced correlation between subjects during the harmonization process due to the entangled batch effects and biological effects (Hu et al., 2023). Therefore, an efficient separation between biological effects (anatomical content) and batch effects (imaging feature biases) and a precise estimation of these effects are imperative.

To address the harmonization problem in neuroimaging data and overcome the existing issues in the harmonization methods, we attempted to utilize the diffusion probabilistic model (Ho et al., 2020), and our contributions are as follows:

- We formulated the neuroimaging harmonization problem as a controllable domain adaptation problem. Domain embedding controlled sampling process diffuses images from batch effect removed domain to the target image domain achieving the multi-domain harmonization.
- We proposed the skip sampling step to reduce the sampling steps for the harmonization purpose.
- We tested the proposed method in two public datasets of ABIDE II and ADNI 1:
  - Our diffusion model-based harmonization method achieved superior harmonization results comparing to GAN based models when using ABIDE II dataset for *site effects harmonization*. We also visually demonstrated the effectiveness of multiple diffusion trajectories learning of the proposed method.
  - We evaluated the *scanner effects harmonization* by downstream perivascular spaces (PVS) segmentation analysis on ADNI 1 dataset and showed improved consistency of the PVS count ratio across scanners.

In this study, we show that denoising diffusion model could effectively address the batch effects harmonization with the preservation of anatomical details. We also found the learned domain invariant condition guarantees more accurate anatomical details of the harmonized images compared with fixed edge map condition. In addition, diffusion model provides strong ability of learning multiple diffusion trajectories controlled by the domain embeddings, which enables the multi-domain harmonization through only one single model.

## 2. Related work

### 2.1. Image harmonization with generative model

The field of generative models has garnered significant interest in recent years due to their remarkable capability to learn the joint distribution. Mostly adopted models such as generative adversarial networks (Goodfellow et al., 2014), variational autoencoder (Doersch, 2016), and probabilistic diffusion model (Ho et al., 2020; Song et al., 2020) also had a great impact in the medical image analysis field. GAN models have been popularly implemented in the neuroimage harmonization task. Chang et al. (Chang et al., 2022) modeled Cycle-GAN (Zhu et al., 2017) to generate unpaired synthetic target scanner style image, then used the original image to perform histogram matching with the synthetic target scanner style image and achieve style harmonization. Yao et al. (Yao et al., 2022) proposed a style – content disentangle GAN to harmonize between the contrast enhanced T1-weighted (T1w) and high resolution T2-w(T2w) MRI. Because batch effects are implicitly defined as style, this method has the risk of incorrectly including biological effects as the style. Zuo et al. (Zuo et al., 2021) proposed a variational autoencoder based model Contrast Anatomy Learning and Analysis for MR Intensity Translation and Integration (CALAMITI). CALAMITI encodes image into low-dimensional style-invariant content representation, then generates target batch image with content representation combined with style information. CALAMITI has been validation on multiple studies and showed its effectiveness, however, CALAMITI requires at least two MRI modalities to learn the batch effect invariant anatomy, which might not be doable in the clinical setting where T1w is the only available modality. Ren et al. (Ren et al., 2021) aimed to remedy the artifacts issue of CycleGAN by proposing a Segmentation-Renormalized (Seg-Renorm) framework with an additional semantic extractor of which the output was used to calculate the trainable scale and shift parameters to normalize each layer of the CycleGAN. However, most of the CycleGAN based generative models only reciprocally translate two sets of domains of images per model. This means that, for the harmonization tasks that involve N domains, there will be a need to train $Combination(N,2)=\frac{N!}{2!(N-2)!}$ CycleGAN models.

Recently, several works have proposed the GAN based methods for multi-domain harmonization. (Liu et al., 2023; Liu et al., 2021) utilized StyleGAN (Karras et al., 2019) architecture to harmonize the multi-domain neuroimage. The latent vector embedded from the target domain sample is used to generate the style code to normalize the layers of generator to generate the output image with target domain style and input image content. Domain style is an umbrella term of the domain-specific imaging feature biases. Because the content was reinforced by a cycle-consistency loss instead of through explicitly defining the separation between content and style, this method faces the challenge of blending styles and content rather than explicitly correct for domain specific biases, producing images that mix features rather than standardize them. (Cackowski et al., 2023) proposed a self-supervised VAE-GAN method called ImUnity. It is trained with the Gamma function manipulated images to simulate the different MRI image contrast across different image domains. At inference stage, a sampled image slice from the target image domain is used to replace the Gamma function manipulated image as reference image to harmonize the input image to the target image domain. However, the Gamma function only manipulates the image contrast and doesn’t change the fine imaging texture (Kassner and Thornhill, 2010) which is one of the technical variabilities exiting in the multicenter dataset. (Roca et al., 2023b) proposed a CycleGAN based method called Image Generation with Unified Adversarial Networks (IGUANe) which has one forward generator to transform N sites images to one reference site. Such universal generator guarantees the content-style disentanglement by only transforming the style without harming the content which shared across N sites. However, training this model with one selected reference image domain involves simultaneously training N+1 generators and 2*N discriminators. If the reference image domain needs to be selected based on the empirical analysis and assuming there are N reference sites, N models need to be trained. In this work, we aim to employ the domain unlearning and conditional generative model to guarantee the consistency of domain invariant anatomical content while harmonizing the multi-domain images in one unified model. We also evaluated the imaging texture harmonization efficacy using radiomics feature analysis.

### 2.2. Domain unlearning

Domain unlearning is an emerging domain adaption technique (Ganin et al., 2016) that shows promise in addressing the challenges of domain bias and data variability in multi-domain image analysis. This approach has been adapted in medical imaging harmonization study and aiMS to create a feature representation that is invariant to specific domain to which the data belongs, while still maintaining the ability to perform the primary task, such as image segmentation or classification (Dinsdale et al., 2021). By posing the problem as a joint domain adaptation task, unlearning methods can effectively remove domain-specific information from the learned features, allowing for better harmonization across different datasets. We implemented an unlearning step to further remove the domain bias in the learned domain invariant feature condition during the model training.

### 2.3. Conditional generative model

Conditional generative model considers the additional information during the generative process and generates samples based on specific conditions or constraints. One of the advantages of conditional generative model is that it allows more controls over the generated samples (Isola et al., 2017). Conditional generative model has been commonly adopted in the multi-model neuroimage analysis (Pan et al., 2021), region of interest (ROI) segmentation (Jung et al., 2023), and neuroimage modality synthesis tasks (Lan et al., 2021; Yang et al., 2018). For our proposed method, a domain invariant condition was designed to guarantee the anatomical details consistency between the original image and harmonized image.

Considering the advantages of the diffusion model, including the stable training (Dhariwal and Nichol, 2021), high-fidelity image generation (Rombach et al., 2022; Saharia et al., 2022), and the ability of learning multiple diffusion trajectories (Biroli et al., 2024), we explored and evaluated the ability of diffusion model in the multi-domain neuroimaging harmonization and proposed a denoising diffusion model-based harmonization framework.

## 3. Proposed method

### 3.1. Multi-domain neuroimaging harmonization as a controllable domain adaption problem

Neuroimaging data acquired at different manufactured scanners, or at different imaging sites often contain non-biological domain shifts introduced by these technical variabilities (Abbasi et al., 2024; Bayer et al., 2022; Hu et al., 2023). We approached the multi-domain neuroimaging harmonization as a controllable domain adaption task (Farahani et al., 2021), which aiMS to learn the batch effects by a domain embedding controlled denoising diffusion process. Since one of the remained challenges in the most neuroimage harmonization techniques is the lack of the ability to disentangle the biological effects and batch effects during the harmonization process (Hu et al., 2023), we conditioned the diffusion process with a learned domain invariant condition. This condition reinforces preservation of the anatomical details shared across image domains during the diffusion harmonization process.

**Fig. 1.**
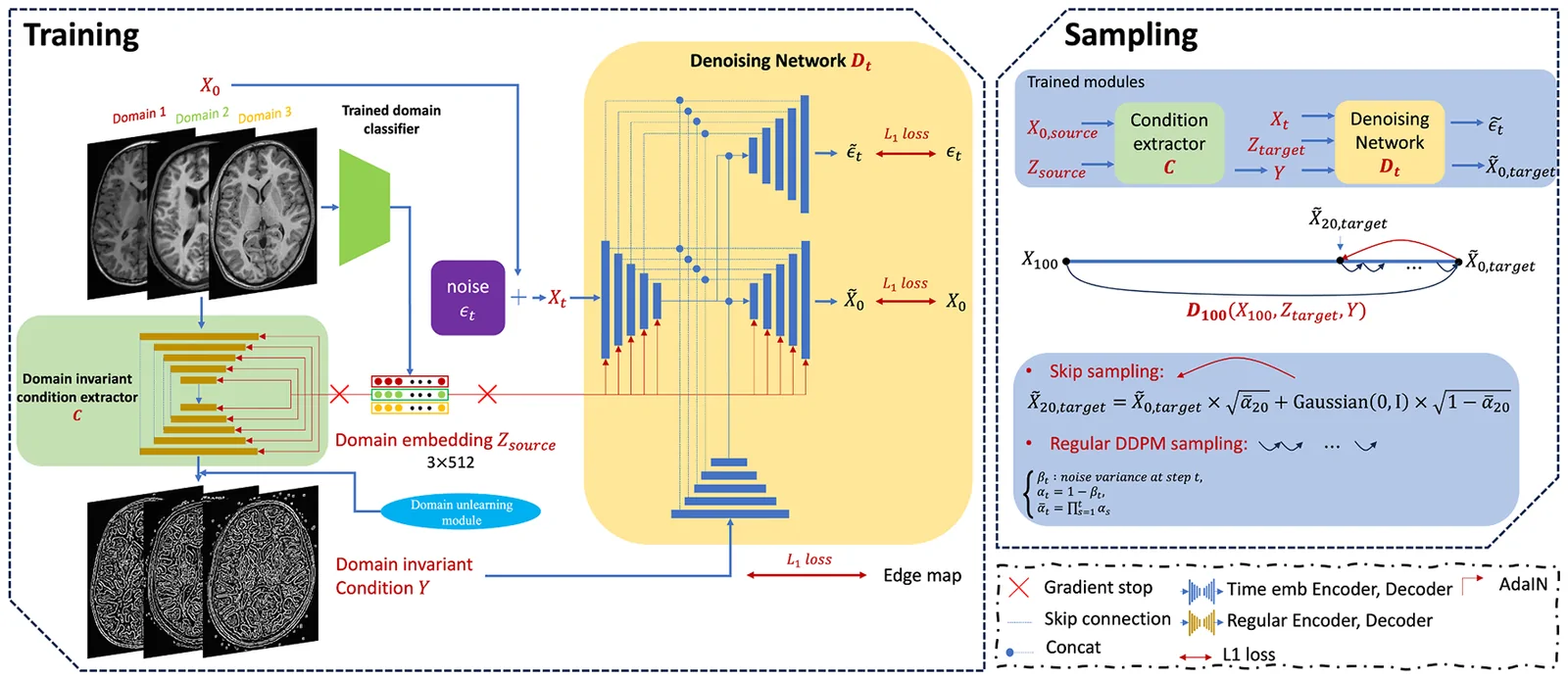
The overall framework of the proposed method with training and sampling details. The proposed harmonization method includes a domain invariant condition extractor ***C*** and a denoising network ***D_t_***, which are both controlled by the domain embedding *Z*.

We introduced a denoising diffusion model based multi-domain harmonization framework (Fig. 1) which includes a domain invariant condition extractor ***C*** and a denoising network ***D_t_***. Both modules were controlled by the domain embedding which determines the diffusion trajectory.

### 3.2. Denoising diffusion probabilistic model

Denoising diffusion probabilistic model (DDPM) has gained a great attention during recent years. DDPM has a strong ability to learn the feature distribution by gradually removing the gaussian noise from the noise corrupted images. During training, DDPM learns to capture the underlying structure of clean images and the noise characteristics at each time step, enabling DDPM to effectively denoise new, unseen images.

The forward diffusion process of DDPM is a Markovian process with T steps, which adds variance *β*_1_, …, *β_T_* scheduled gaussian noise to the image step-by-step to corrupt the image until it becomes the completely random noise. At step *t* the conditional probability of the Markov chain is modeled as *q*(*X*_*t*+1_∣*X_t_*) and the overall forward process is modeled as follows:

(1) $$q(X_{1:T}|X_{0})≔\prod_{t=1}^{T}q(X_{t}|X_{t-1}),$$

(2) $$q(X_{t}|X_{t-1})≔Normal\left(X_{t};\sqrt{1-\beta_{t}}X_{t-1},\beta_{t}I\right),$$

where *X*_0_ is the clean image in the manifold and *X_T_* is the random sample following standard normal distribution. The reverse process is also Markovian and modeled by an approximation distribution *P_θ_*

(3) $$P_{\theta }(X_{0:T})≔P(X_{T})\prod_{t=1}^{T}P_{\theta }(X_{t-1}|X_{t}),$$

(4) $$P_{\theta }(X_{t-1}|X_{t})≔Normal(X_{t-1};\mu_{\theta }(X_{t},\;t),\xi_{\theta }(X_{t},\;t)),$$

where a neural network model learns the reverse distribution *P_θ_*(*X*_*t*−1_∣*X_t_*) by estimating the scheduled gaussian noise at time step t in the *X_t_*. This neural network model can be interpreted as an equally weighted sequence of noise estimation autoencoders *ϵ_t_*(*X_t_*). The training objective of the DDPM is:

(5) $$L=E_{x,\;\epsilon ∼Normal(0,1)}[{\left\|\;\epsilon -\epsilon_{t}(X_{t})\right\|}^{2}],$$

where *ϵ* is the standard gaussian noise added to *X_t_*. The *ϵ* prediction parameterization exhibits similarities to Langevin dynamics and simplifies the variational bound of the diffusion model to an objective that resembles denoising score matching (Song et al., 2020). DDPM serves as a strong generative model to learn the domain variant distribution in our proposed harmonization method.

### 3.3. Learned domain invariant conditioned controllable denoising diffusion model

The denoising network ***D_t_*** was implemented following DDPM paper which is a positional time embedding *t* controlled U-Net (Ronneberger et al., 2015) model with slight modifications. ***D_t_*** has two encoders and two decoders. As shown in Fig. 1, the encoder receiving the noise corrupted image *X_t_* is controlled by the domain embedding, while the encoder taking the domain invariant condition *Y* is not controlled; the decoder predicting the uncorrupted image *X*_0_ is controlled by the domain embedding, while the decoder estimating the standard gaussian noise ${\tilde{\epsilon }}_{t}$ is not controlled. The rationale of this design is due to domain invariant nature of *Y* and ${\tilde{\epsilon }}_{t}$.

The domain invariant condition extractor ***C*** is a regular U-Net model which are controlled by the domain embedding and is regarded as a function to project images from different image domains to the same domain invariant condition domain. To correlate the domain embedding with the denoising network and the condition extractor, we utilized Adaptive Instance Normalization (AdaIN) (Huang and Belongie, 2017) to normalize each layer in the target layers of the modules.

The loss function of the proposed method is as follow:

(6) $$Y=C(X_{0},\;Z_{source}),$$

(7) $${\tilde{\epsilon }}_{t},\;{\tilde{X}}_{0}=D_{t}(X_{t},Z_{source},Y),$$

(8) $$L_{noise}=|{\tilde{\epsilon }}_{t}-\epsilon_{t}|,$$

(9) $$L_{recon}=|{\tilde{X}}_{0}-X_{0}|,$$

(10) $$L_{cond}=|Y-canny\;edge|,$$

(11) $$L_{total}=L_{noise}+L_{recon}+L_{cond},$$

where ***D_t_*** is the denoising network at time step *t*, which outputs the estimated noise ${\tilde{\epsilon }}_{t}$ and predicted input data ${\tilde{X}}_{0}$; ***C*** is the domain invariant condition extractor; *Z_source_* is the domain embedding of ${\tilde{X}}_{0}$. Instead of using *L*_2_ loss in the DDPM implementation, we used *L*_1_ loss which has been reported with superior image quality in the image generation (Zhao et al., 2016).

As shown in Fig. 1, *L_noise_* and *L_recon_* update the denoising network ***D_t_***, while all three components of the *L_total_* update the domain invariant condition extractor ***C***. Three components of the loss function reinforce the condition extractor to extract the condition which is domain invariant and is imbued with enough anatomical details for the domain embedding controlled denoising network to estimate the noise at step t and predict the original input data. For our experiments which focus on imaging texture harmonization, canny edge detector generated edge map was a good ground truth to calculate *L_cond_*. We applied the canny edge detector from the Scikit-image image processing library (der Walt et al., 2014).

We trained an independent image domain classifier and used its layer with 512 elements before the classification head as the domain embedding *Z_source_* (Fig. 1). Since domain classifier is separated from the overall framework, training won’t update the classifier.

#### 3.3.1. Unlearning step

To further remove the remaining domain variant features in the learned condition, we incorporated a domain unlearning adversarial loss inspired by Dinsdale et al. (Dinsdale et al., 2021). Unlearning process is an adversarial learning process where the domain invariant feature extractor *C* tries to generate most domain invariant features which are judged by the discriminator *disc*. The reached Nash equilibrium (Nash, 2024) between *C* and *disc* indicates a balance between the adversarial training, and the extracted features *Y* are domain invariant.

The *disc* is a five-layer U-Net encoder and serves as a domain classifier. The loss function of *disc* is the multi-class cross entropy loss defined as follows:

(12) $$L_{disc}(Y,\;disc;\;\Theta_{disc})=-\frac{1}{N}\;\sum_{n=1}^{N}log\frac{Exp(Y_{n,\;domain_{n}})}{\sum_{q=1}^{Q}Exp(Y_{n,\;q})},$$

where *Y_n, q_* is the output logit of Discriminator; *N* is the number of samples of one batch; *Q* is the number of the domains; *domain_n_* is the actual domain where subject *n* belongs to; Θ*_disc_* is the set of parameters of *disc* to be updated. The *disc* forces feature extractor *C* to generate most domain invariant features by an adversarial training process. These features are used as domain invariant conditions for the diffusion process to generate images with target image style, thereby achieving harmonization. The unlearning loss function (13) for the domain invariant feature extractor *C* and the final form of the diffusion loss function are defined as follows:

(13) $$L_{unlearning}(Y,\;disc;\;\Theta_{C})=-\frac{1}{N}\;\sum_{n=1}^{N}\frac{1}{Q}\;\sum_{q=1}^{Q}\frac{Exp(Y_{n,\;q})}{\sum_{q=1}^{Q}Exp(Y_{n,\;q})},$$

(14) $$L_{final}=L_{total}+L_{unlearning},$$

where Θ*_C_* is the set of parameters of feature extractor *C* to be updated. During the training, *L_disc_* was calculated and backpropagated prior to the *L_final_* and both loss terMS were updated iteratively. We also observed that using the *disc* initialized with pre-trained weights which classifies the domain of learned condition would reduce the number of epochs required for unlearning. This was also reported in the Dinsdale et al’s work (Dinsdale et al., 2021). The unlearning loss forces the feature extractor *C* to generate the most domain invariant features which confuses the discriminator to predict equal probabilities across all domains.

### 3.4. Skip sampling and inference

At the training stage, assume we have 100 steps for the forward process to corrupt the original image *X*_0_ to a random noise image *X*_100_ by adding gaussian noise *ϵ_t_* gradually. During the reverse process, at time step t, the denoising network ***D_t_*** estimates the noise *ϵ_t_* in *X_t_* given the domain invariant condition *Y* and controlled by the domain embedding *Z_target_*. At the inference stage, a successfully trained diffusion model would generate the target domain style image with domain invariant content through a reverse process controlled by *Z_target_*. To reinforce the content consistency, ***D_t_*** is also trained to predict the uncorrupted image *X*_0_ given *X_t_* at each time step *t* besides estimating the noise *ϵ_t_*.

Because *X*_100_ is the random noise image, at diffusion time step 100, the domain style prediction of *X*_0_ is purely controlled by *Z_target_* and the anatomical prediction of *X*_0_ is purely conditioned on domain invariant condition *Y*. One point to note, *Z_source_* was used for the training of ***D_t_*** during the training process (as shown in Fig. 1), however, at the inference step *Z* could be any target domain embedding.

(Biroli et al., 2024) Revealed three distinct dynamic regimes of the reverse generative diffusion process. In regime I, the diffusion trajectories are fluctuating and inseparable across image domains; in regime II, the ‘speciation’ unravels the gross structure of data belong to different image domains; in regime III, trajectories collapse to specific sample points within the distributions of different image domains. We observed similar dynamic behavior in our proposed conditional diffusion model and designed the inference process specifically to skip the early regimes. Because the early regimes have highly correlated diffusion trajectories, skipping such steps improves the inference speed without compromising the controllability of diffusion trajectories. The key to the sampling step for the harmonization is that the target domain ${\tilde{X}}_{0,target}$ (real T1w image) is predicted at step 100 (random noise image), then we corrupt the ${\tilde{X}}_{0,target}$ back to ${\tilde{X}}_{20,target}$ (see Fig. 1). For this step we call it skip sampling. After the skip sampling, we use regular DDPM sampling to sample the image from ${\tilde{X}}_{20,target}$ to ${\tilde{X}}_{0,target}$ (see Algorithm 1). Since direct prediction of ${\tilde{X}}_{0,target}$ from *X*_100_ still misses details of target domain style, by doing skip sampling we could shorten the DDPM sampling process and generate images with rich target domain style as shown in Fig. 3. Selection of the number of steps for the skip sampling is based on the empirical study of the given dataset.

**Table:** 

Algorithm 1
$$\begin{array}{cc} \; & \;X_{0,\text{source}}\;:\mathrm{Input}\;\mathrm{image}\;,\;Z_{\text{source}}\;:\mathrm{Source}\;\mathrm{domain}\;\mathrm{emb},\;Z_{\text{target}}\;:\\ \; & \;\;\;\mathrm{Target}\;\mathrm{domain}\;\mathrm{emb}\\ \; & \;\;X_{100}∼\text{Gaussian}(0,\;I)\\ \; & \;\;Y=C(X_{0,\text{source}},\;Z_{\text{source}})\\ \; & \;\;{\tilde{X}}_{0,\text{target}},\;{\tilde{\epsilon }}_{100}=D_{100}(X_{100},Z_{\text{target}},Y)\\ \; & \;\;\text{skip}=20\\ \; & \;\;X_{t}={\tilde{X}}_{0,\;\text{target}}\times \sqrt{{\bar{\alpha }}_{\text{skip}}}+\text{Gaussian}(0,I)\times \sqrt{1-{\bar{\alpha }}_{\text{skip}}}\mathrm{Skip}\;\mathrm{sampling}\\ \; & \;\;\text{for}\;t=\text{skip},\ldots ,1,\mathrm{do}\\ \; & \;\;\;\;\;z∼\text{Gaussian}(0,\;I)\;\text{if}\;t>1,\;\text{else}\;z=0\\ \; & \;\;\;\;\;{\tilde{X}}_{0,\text{target}},\;{\tilde{\epsilon }}_{t}=D_{t}(X_{t},Z_{\text{target}},Y)\\ \; & \;\;\;\;\;X_{t-1}=\frac{1}{\sqrt{\alpha_{t}}}\left(X_{t}-\frac{1-\alpha_{t}}{\sqrt{1-\bar{\alpha_{t}}}}*{\tilde{\epsilon }}_{t}\right)+0.5*\log(1-\alpha_{t})*z\mathrm{DDPM}\;\mathrm{sampling}\\ \; & \;\;\text{end for}\\ \; & \;\;\text{return}\;X_{0} \end{array}$$

#### 3.4.1. 3D fusion module

In-between-slice difference of the harmonized 3D data has been acknowledged by the recent work as a limitation of 2D harmonization method (Hu et al., 2023; Lin et al., 2024). To overcome this challenge which harMS the quality of harmonized images, we trained additional 3D fusion module to fuse harmonized 2D slices into a 3D data to remove the potential in-between-slice error inspired by (Zuo et al., 2021).

The fusion network is a residual convolutional network (ResNet) (He et al., 2016) without any down-sampling layers which was designed to maintain image details not being compromised during the fusion process. The skip connections between early layers and late layers were implemented. The input of the fusion network is the stack of 2D harmonized images across axial, coronal, and sagittal directions, which means the input image has three channels. We used the training samples of the harmonization tasks where source image domains are the same as the target image domains, in other words, fusion network training is a supervised learning process. The 3D fusion module architecture and visual examples can be found in supplementary Fig. S1 and S2.

## 4. Experiments and results

### 4.1. Dataset

#### ADNI 1:

We selected the older healthy participants of the Alzheimer’s Disease Neuroimaging Initiative (ADNI 1) (Jack Jr et al., 2008) cohort (adni.loni.usc.edu) as one of the experiment datasets. The ADNI was launched in 2003 as a public-private partnership, led by Principal Investigator Michael W. Weiner, MD. The primary goal of ADNI has been to test whether serial magnetic resonance imaging (MRI), positron emission tomography (PET), other biological markers, and clinical and neuropsychological assessment can be combined to measure the progression of mild cognitive impairment (MCI) and early Alzheimer’s disease (AD). ALL ADNI study procedures were conducted in accordance with the Declaration of Helsinki and approved by the Institutional Review Boards (IRB) of all participating institutions. Written informed consent was obtained from all participants or their authorized representatives. Only de-identified data were used in this study. 456 participants, aged between 55 and 90 years, were gathered during the 12-month follow-up period after recruitment. All 456 subjects’ MRI images collected for this study follow the same image acquisition: T1w MPRAGE MRI images using scanners manufactured by either GE Healthcare, Philips Medical SysteMS, or Siemens Medical Solutions operating at 1.5T field strength. These images had a voxel resolution of 1.25 × 1.25 × 1.2 mm3 and were obtained with the following acquisition parameters: TR=2400 MS, TE=3.6 MS, flip angle=8.0 degrees, FOV=24 cm. We performed visual image quality check and scans with poor data excluded from analyses. Poor scan quality was characterized by T1w volumes with excessive head motion, reduced grey matter (GM)/ white matter (WM) tissue contrast, unidentifiable anatomy, blurred images and salt-and-pepper noise, which obstructed the visibility of PVS.

#### ABIDE II:

We selected data of four sites from the Autism Brain Imaging Data Exchange (ABIDE II) (Di Martino et al., 2014) as one of the experiment datasets including 48 subjects from Erasmus University Medical Center Rotterdam (EMC), 36 subjects from ETH Zurich (ETH), 90 subjects from Georgetown University and 78 subjects from NYU Langone Medical Center (NYU). Detailed acquisition parameters could be found in the Table 1. All contributing sites obtained approval from their respective IRB and written informed consent from participants or their legal guardians. Only de-identified data were shared and used for secondary analysis in accordance with ethical guidelines.

FreeSurfer version 7.1.1 recon-all module on the Laboratory of Neuro Imaging (LONI) pipeline system (https://pipeline.loni.usc.edu) initially resampled all images to 256 × 256 × 256 image size, 1 × 1 × 1 mm3 voxel resolution, rescaled intensity range to 0-255, and reoriented the images to RAS (Right, Anterior, Superior) coordinate system. Non-parametric Non-uniform intensity Normalization (N3) was used to correct the bias field through FreeSurfer. The bias filed correction using N3 was only meant to correct the bias field present equally in images acquired from different scanners or imaging sites. Preprocessed images have voxel intensity rescaled to 0-255, which removed the intensity value range batch effect observed from images acquired from different sites and scanners. Then we selected axial slices from each subject with brain signals (whole background signal slices were discarded) which resulted in 45600 training samples/11400 testing samples for ADNI 1; 35280 training samples/8820 testing samples for ABIDE II.

### 4.2. Perivascular spaces

PVS (Barisano et al., 2022; Wardlaw et al., 2020), also known as Virchow-Robin spaces, are fluid-filled spaces that surround penetrating blood vessels in the brain. These spaces are lined by a layer of cells and contain cerebrospinal fluid (CSF). PVS play an important role in the brain waste clearance exchanging nutrients and waste products between CSF and brain tissue. They are more visible in T2-weighted MRI sequences, in comparison with T1-weighted sequences. In certain conditions or diseases, PVS may become enlarged or show abnormalities, which can be indicative of underlying health issues. Frangi filter (Frangi et al., 1998) is an image processing algorithm that detects tubular structures in the 2D or 3D image spaces and commonly adopted to segment PVS in the T1w or T2w MRI images (Ballerini et al., 2018; Lan et al., 2023; Sepehrband et al., 2019). To generate the PVS count, binarized PVS map would be generated at first. Then the regions with less than 3 connected voxels would be removed, which could be potential random noise or motion corruption captured by Frangi filter. Finally, the overall PVS count would be divided by total white matter volume to generate the PVS count ratio as a more generalized measure. We used PVS count ratio as an evaluation metric of scanner effects existing in ADNI 1.

Batch effects in the T1w images from ADNI 1 are the scanner effects and we focused on the PVS segmentation as the downstream analysis to demonstrate the harmonization efficacy. Batch effects in the T1w images from ABIDE II are the site effects and we reported Fréchet inception distance (FID) score (Heusel et al., 2017) and qualitative visualization to demonstrate the harmonization efficacy.

### 4.3. Radiomics analysis

Radiomics is a rapidly evolving field in medical imaging that involves extracting quantitative features from medical images to provide additional information for clinical decision-making (McCague et al., 2023). This approach aiMS to capture tissue characteristics such as heterogeneity, which may be difficult to recognize or quantify by the human eye. Harmonization techniques can benefit from radiomics analysis as an additional quantitative measurement of the imaging texture change after the harmonization, particularly in multicenter studies where variability in scanner models and imaging protocols can affect data integration ability (Hajianfar et al., 2024).

We extracted a total of 93 radiomics features from the WM and GM respectively of each 3D image and each 3D harmonized image using the IBSI-compatible PyRadiomics package in Python (Van Griethuysen et al., 2017). These features comprised two sets: 18 first-order features and 75 texture features, which included (i) 24 Gray Level Co-occurrence-Matrix (GLCM), (ii) 16 Gray Level Run Length Matrix (GLRLM), (iii) 14 Gray Level Dependence Matrix (GLDM), (iv) 16 Gray Level Size Zone Matrix (GLSZM), and (v) 5 Neighboring Gray Tone Difference Matrix (NGTDM). The number of bins for the PyRadioimcs feature extraction was set at 32 for both WM and GM analysis.

To visualize the distribution shift of the radiomics features after the harmonization in the low dimension space, we employed t-distributed stochastic neighbor embedding (t-SNE) (der Maaten and Hinton, 2008) as our data exploratory technique.

### 4.4. Experimental setup

The denoising network was designed following original DDPM U-Net noise estimation model with 5 skip-connection levels and each level was constituted by two ResNet (He et al., 2016) blocks each with two convolutional layers. Self-attention (Vaswani et al., 2017) was calculated at down-sampling and up-sampling path at third level and bottleneck layers. The condition extractor was designed the same way as the denoising network with only one encoder and one decoder and not controlled by the positional time embedding. Noise variance *β* was quadratically scheduled between 0.0001 and 0.5 with 100 steps as a fixed sequence. CycleGAN and Seg-Renorm models were downloaded from their public GitHub repositories and finetuned accordingly with our experimental datasets. We have made necessary modifications such as changing the number of input and output channels from 3 to 1 to adapt CycleGAN model from originally processing RGB images to gray scale MRI images to guarantee its best performance on our experimental datasets. All experiments were implemented and conducted using Python 3.8 and PyTorch 1.13.1.

The separately trained domain classifier is a ResNet with 6 levels and 2 convolution layers at each level, with self-attention before the sixth level. The domain classifier has been trained to classify the corresponding image domain and we used its multilayer perceptron layer with 512 elements before the classification head as the domain embedding. All models were trained and reached optimal results on the validation data.

### 4.5. Domain embedding controls DDPM to learn multiple diffusion trajectories

**Fig. 2.**
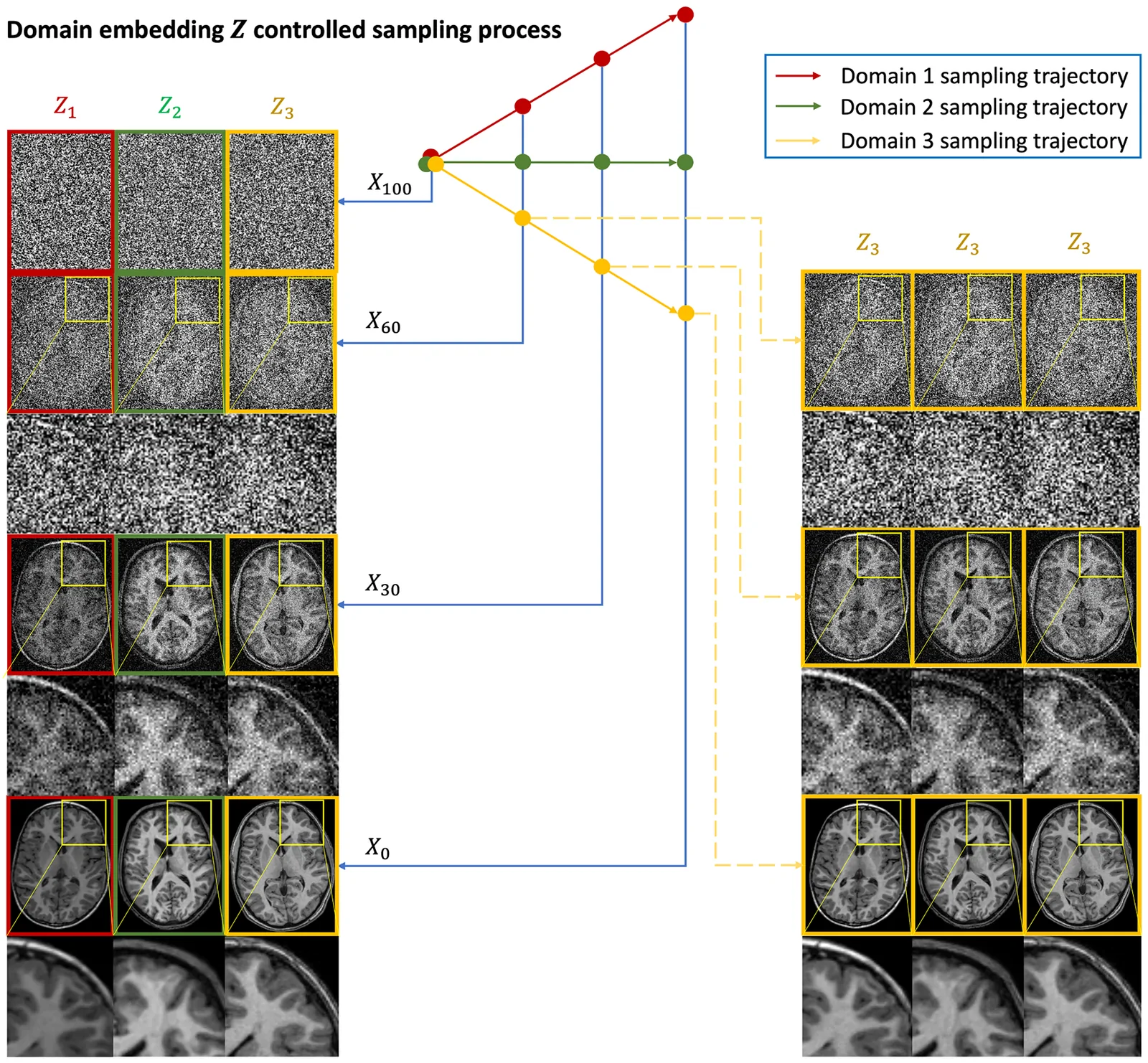
Visual demonstration of the learned trajectories. Domain embedding ***Z*** steers the sampling trajectories achieving the multi-domain harmonization. *X*_100_ ~ *Gaussian*(0, *I*) as shown in the Fig.. On the left side of the Fig., *Z_target_* = *Z_source_* and site difference could be visually seen at each sampling step; on the right side of the Fig., *Z_target_* = *Z*_3_, which means all three image samples were harmonized to domain 3. Comparison between left and right side of the Fig. demonstrates the effectiveness of harmonization at each sampling step.

Domain embedding controls diffusion model to learn multiple diffusion trajectories at once which enables multi-domain harmonization with single model. Fig. 2 is the qualitative visualization of the diffusion sampling process for the ABIDE II dataset. One sample of each of the three sites was randomly selected for this visualization. To demonstrate multiple intermittent steps of the diffusion process of the harmonization task, skip sampling was not used. *Z*_1_ (red), *Z*_2_ (green) and *Z*_3_ (yellow) represent the three domain embeddings generated by the pretrained domain classifier and colored arrows represent different sampling trajectories. Samples from four time-steps *X*_100_, *X*_60_, *X*_30_, *X*_0_ are shown for visual comparison. For the images on the left side, *Z_target_* equals to *Z_source_*; for the images on the right side, *Z_target_* is *Z*_3_. As time *T* goes from 100 to 0, the site difference (approximately indicated by the distance between each pair of dots at each time step) is increasing, which can also be visually verified by image samples’ differences at each time step on the left side. This indicates that the denoising network learned different diffusion trajectories and correctly correlated to the control domain embedding. By controlling sampling process with *Z*_3_ on the right side, the image samples difference at each time steps were reduced resulting in harmonized images.

### 4.6. Evaluation of harmonization on ABIDE II

**Fig. 3.**
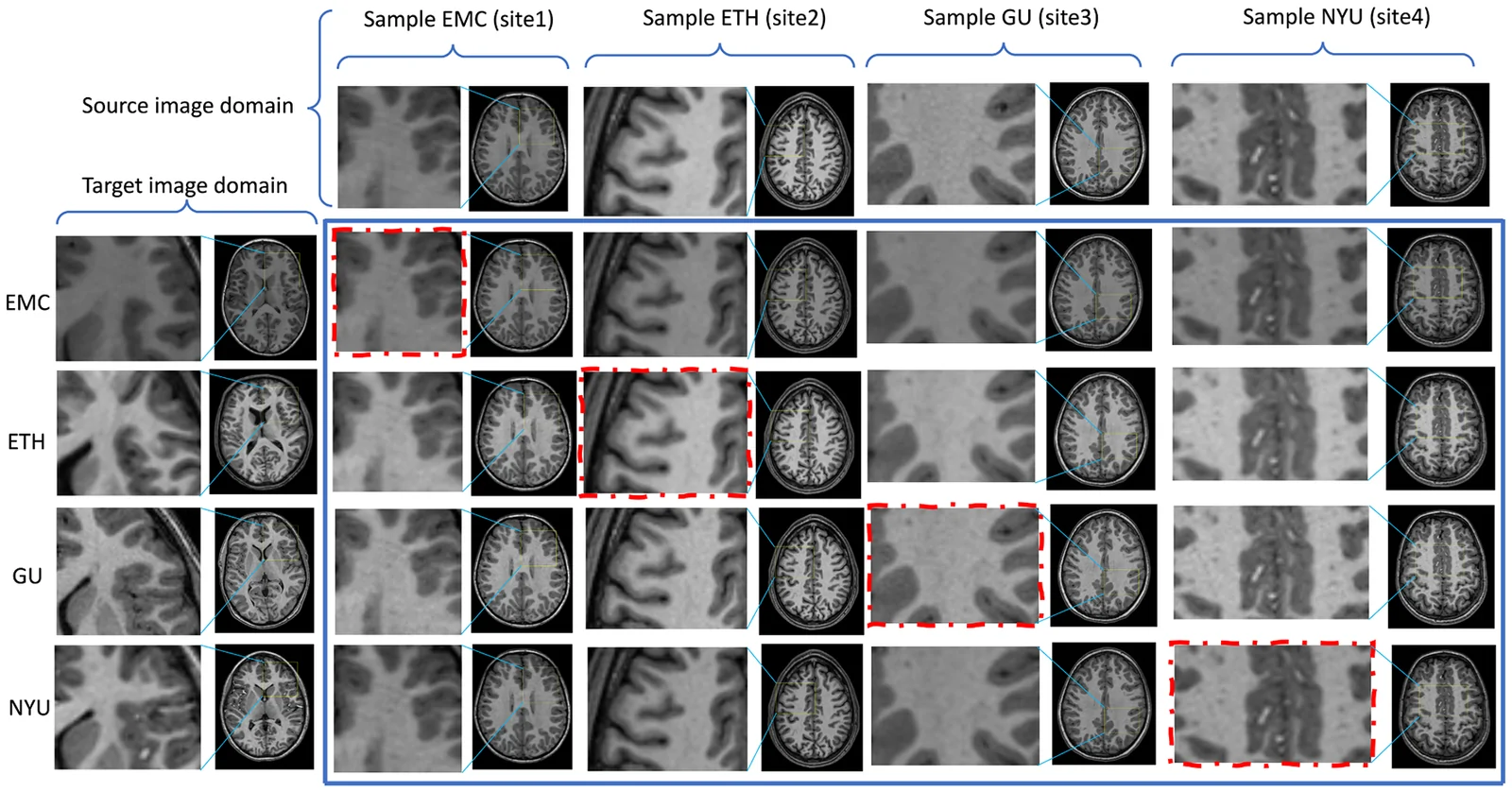
Qualitative results of the harmonization on ABIDE II. The far-left column shows samples of target image domains (reference of radiomic texture), and the top row shows samples of source image domain (reference of anatomical details). The red dash squares highlight the harmonization case where target image domain equals to the source image domain. It could be easily noted that the source images have been harmonized to the target image domain with corresponding textures and contrasts and have anatomical details preserved.

Fig. 3 shows the harmonization results of image samples from four sites EMC, ETH, GU and NYU of ABIDE II to the target image domains respectively. The far-left column shows samples of target image domains (reference of imaging texture), the top row shows samples of source image domain (reference of anatomical details), and the rest of the images show harmonization results to the corresponding target image domain. The red dash squares highlight the harmonization case where target image domain equals to the source image domain. Within the zoomed in regions, it could be easily noted that the source images have been harmonized to the target image domain with corresponding textures and contrasts and have anatomical details preserved.

In order to quantitatively evaluate our proposed method and compare with other state-of-the-art GAN based unpaired harmonization methods, we calculated the FID scores and reported in Table 2. We evaluated two baseline models CycleGAN and Seg-Renorm on ABIDE II with four sites data included. Since both CycleGAN and Seg-Renorm harmonize two sites’ data per model, we trained 6 models for Cycle-GAN and 6 models for Seg-Renorm to harmonize all four sites’ data correspondingly. Similar to the original Seg-Renorm paper, FreeSurfer 7.1.1 (Fischl, 2012) was used to generate the volumetric segmentation mask for the training purpose of Seg-Renorm as indicated in the original paper. We reported four sets of FID scores with target image domain versus other three image domains as shown in Table 2, and InceptionV3 (Szegedy et al., 2016) was used as the feature extractor to calculate the FID score instead of a customized feature extractor to avoid any dataset induced biases. All three harmonization methods have FID scores reduced compared to the FID scores among the original images, which means that after the harmonization, harmonized images are perceptually more similar as the images from the target image domain. Proposed method had the best FID score for three harmonization cases and tied with CycleGAN at the harmonization case of the EMC site.

**Fig. 4.**
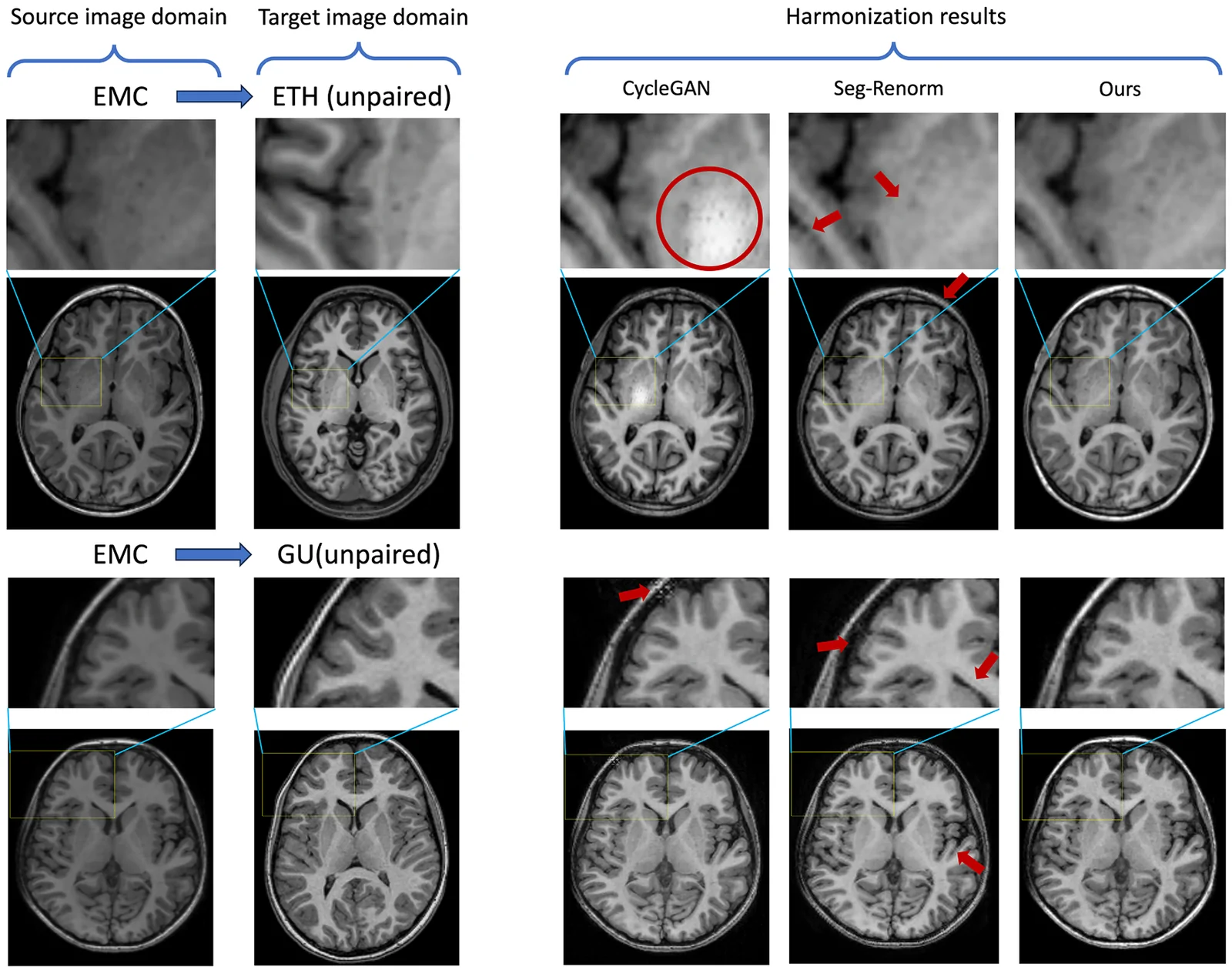
Qualitative comparison with baseline models. First two columns show samples from source image domain (reference of anatomical details) and unpaired sample from target image domain (reference of the radiomic texture). Red arrows indicate the areas with generated artifacts and mild stripe patterns which are not present in both source and target image domain. Proposed method generated the most anatomical details accurate and radiomic texture aligned harmonization results.

Fig. 4 shows qualitative evaluation of the harmonization results from all three methods. First two columns show samples from source image domain (reference of anatomical details) and unpaired sample from target image domain (reference of imaging texture). We noticed that CycleGAN has universal artifacts present in the generated images and might not be able to be correctly quantified by the FID score. Seg-Renorm was able to solve the universal artifacts issue in the CycleGAN generated images, however, it still contains mild stripe patterns which are not present in the source images. Additionally, there are mild anatomy distortion presented in the Seg-Renorm results (indicated by the red arrows). Proposed method generated the most anatomical details accurate and imaging texture aligned harmonization results.

**Fig. 5.**
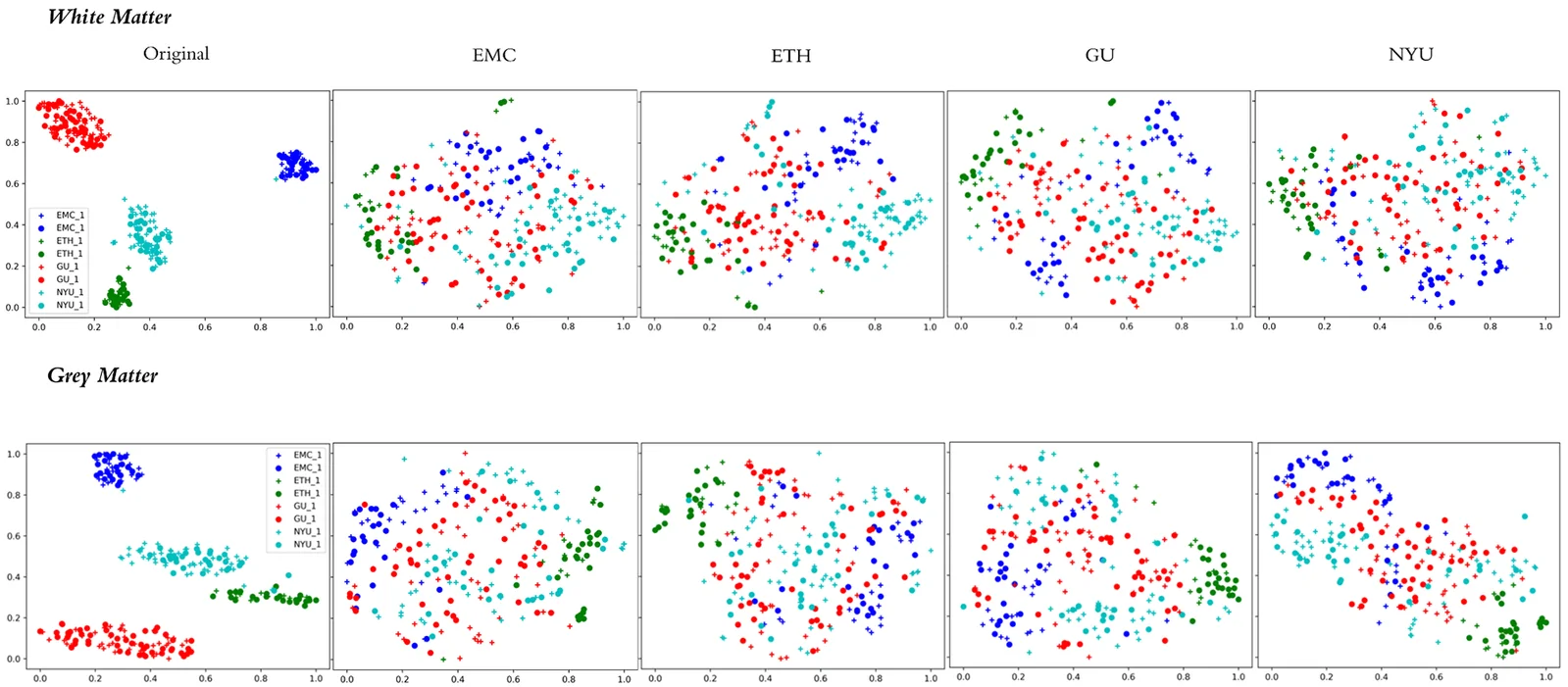
t-SNE visualization of harmonization effects on radiomics feature clustering. t-SNE plots of radiomics feature (93 features extracted from each 3D image) distributions in original versus harmonized image spaces. Colors of the markers represent different imaging sites. Shapes of the marker represent subject’s diagnosis with ASD (solid circle) and control (solid cross). Radiomics analysis for white matter region and grey matter region were conducted independently. Distinct radiomics feature clusters are evident in the original image space, whereas this separation becomes significantly reduced in the harmonized image spaces.

Fig. 5 shows the t-SNE plots of the radiomics feature distributions in the original image space, and the radiomics features distributions in the corresponding harmonized image spaces. Clear radiomics feature clusters can be identified in the original image space. However, such separation became significantly reduced in the harmonized image spaces, indicating the effectiveness of the harmonization process. We also observed similar harmonization effects on both ASD diagnosed subjects and control subjects from Fig. 5. Note that the t-SNE plots of harmonized images also present some local small clusters, which could potentially be explained by the demographic information such as age and sex, and the difference of the sample size. For example, ETH data includes the subjects aged between 14-31 years old, which is outside of the age range of EMC data (6-11 years old) and GU data (8.1-13.9 years old), and partially overlapped with NYU data (5.2-34.8 years old).

**Fig. 6.**
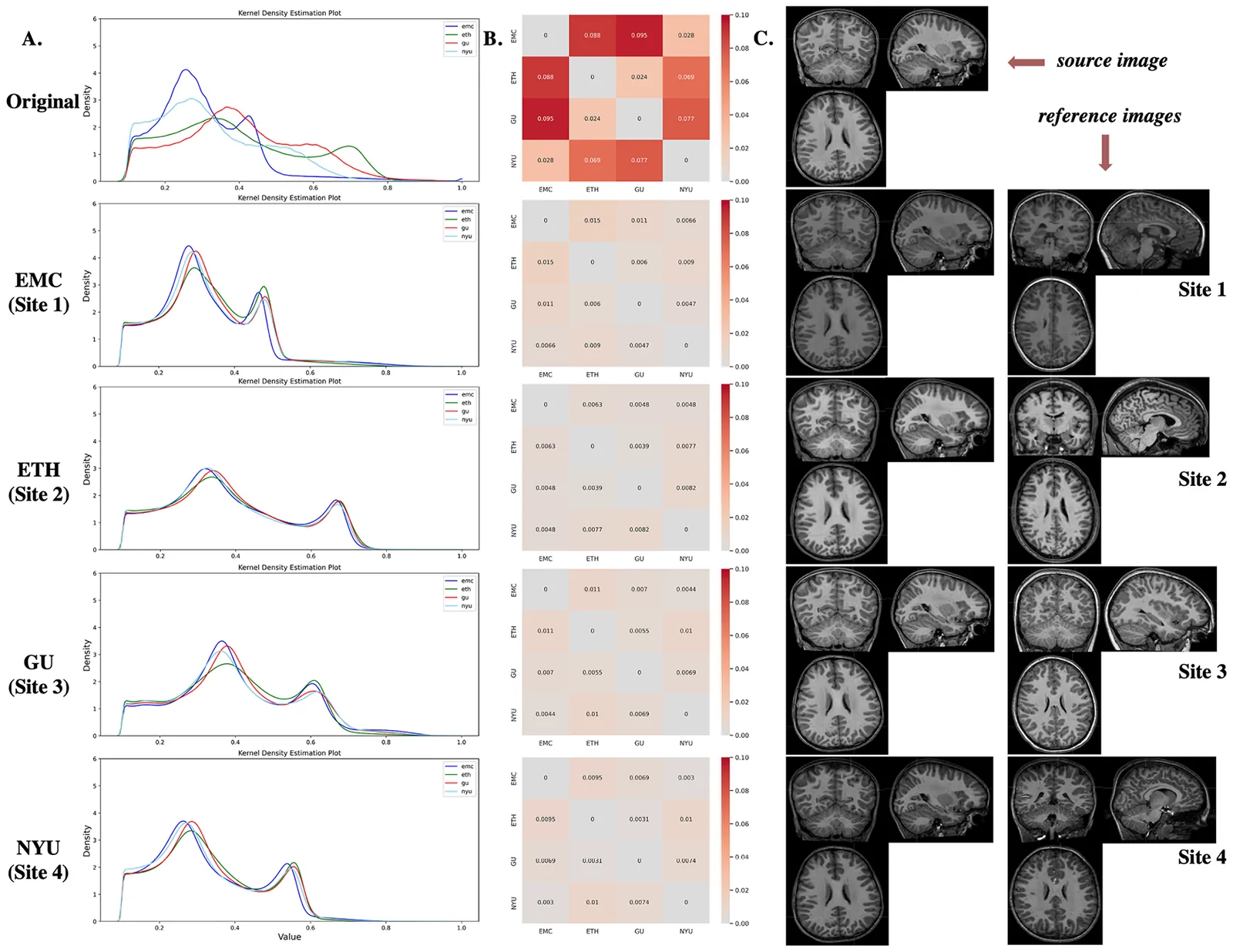
Harmonization quantification through the voxel intensity density plot and Wasserstein Distance (WD), along with 3D visualization of the harmonized images. A. the density plots of original and harmonized (four target sites) images from four imaging centers. Density curves for the harmonized images indicate improved similarity to the target domain. B. the pairwise Wasserstein Distance (WD) metric, revealing that pairwise voxel intensity distribution similarities significantly increase post-harmonization, reflecting effective harmonization. C. an example of original image and fused harmonization results compared with reference images, demonstrating visually enhanced similarity to target domain samples and removed in-between-slice differences.

To quantify the similarity of the voxel intensity distribution for images acquired from different imaging centers and for harmonization effect in ABIDE II dataset, we used voxel intensity density plot (Silverman, 2018) and Wasserstein Distance (WD) (Panaretos and Zemel, 2019). As shown in Fig. 6, column A shows density plots of the original images from four imaging center, followed by 4 plots with density curves of images harmonized to the target image domain. The fused harmonized 3D images from all testing data were used to generate the density plots. Column B shows the WD metric among the original images and harmonized images. The smaller WD indicates more similar voxel intensity distributions. After the harmonization, all pairwise voxel intensity distribution similarities significantly increased indicating effective harmonization process. Column C shows one example with fused harmonization results compared with the target domain samples for the visual inspection. Successfully trained fusion network removed the in-between-slice difference of the harmonized 3D images as shown in Column C.

To further quantify the harmonization effects on the ABIDE II dataset and evaluate the anatomical consistency between the original images and harmonized images, a volumetric segmentation of WM (red), GM (yellow), and cerebral spinal fluid (CSF) (dark blue) was conducted. We used FMRIB’s Automated Segmentation Tool (FAST) (Zhang et al., 2001) to segment the three anatomical regions. FAST algorithm accounts for the mixed tissue contributions at the boundaries of WM, GM, and CSF to reduce the segmentation errors caused by the partial volume effects.

**Fig. 7.**
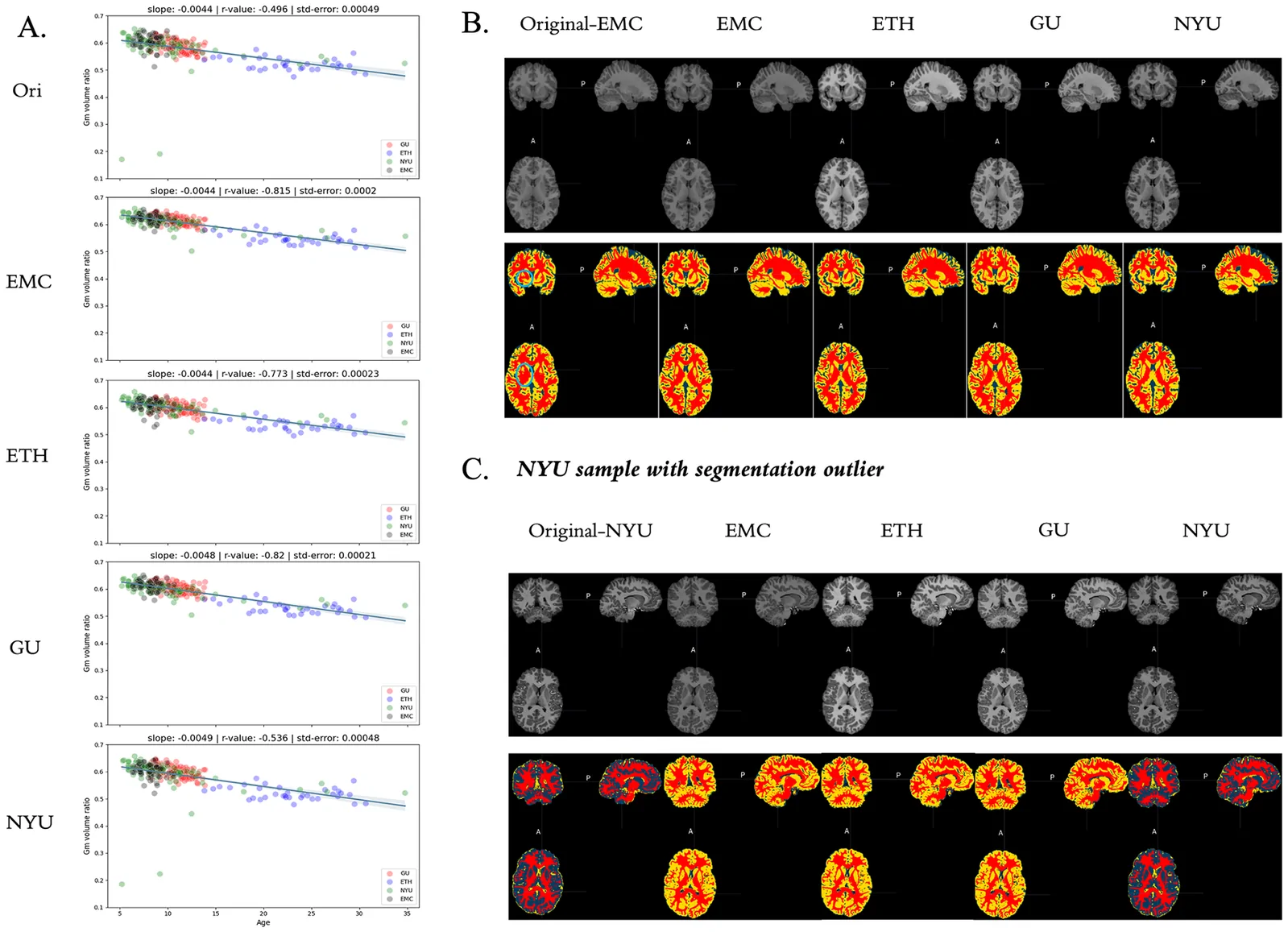
Volumetric analysis of harmonization effects. A. The correlation between age and grey matter volume ratio (divided by the total volume of brain parenchyma) of original image (Ori) and harmonized images respectively. A regression line was plotted for each scatter plot with R-value and standard error indicated in the plot title. B. A visual example of the white matter (red) and grey matter (yellow) segmentation using the brain tissue segmentation pipeline FAST from original image and harmonized images respectively. C. A visual example of segmentation outlier of a NYU sample (the grey matter was falsely segmented as cerebral spinal fluid from original NYU sample).

We focused on the volumetric analysis of GM region which is subject to significant changes across the lifespan (Taki et al., 2011). As shown in Fig. 7 A. a negative linear relationship between age and GM volume ratio (divided by the total volume of brain parenchyma) is captured by the linear least square regression line in the original dataset (ORI) including images from the 4 imaging sites: EMC (grey), ETH (blue), GU (red), and NYU (green). Such negative linear relationship became stronger after the harmonizations to four reference sites respectively indicated by the regression lines with steeper slope, larger absolute value of R-value and smaller standard error, which demonstrates the reduced GM volume ratio variance introduced by the image domain biases and guaranteed anatomical consistency between original and harmonized images.

One segmentation result of an EMC sample is presented in Fig. 7 B. showing the segmentation form the original image and from four reference domain harmonized images respectively. We also observed that the harmonized image (second column) with the same refence and original domain can produce better segmentation result then its original image indicated by blue circles in the first column segmentation result.

Fig. 7 C. shows one segmentation outlier of the NYU sample where the GM region was falsely segmented as CSF. Such outliers can be also observed from two points at the bottom left corner of Ori and NYU plots (Fig. 7 A). However, the harmonized results to other three reference sites didn’t produce this type of segmentation error evidenced in the rest of the plots of Fig. 7 A., which demonstrates one of the necessities of the image harmonization when certain domain imaging feature bias is susceptible to the automatic segmentation pipeline error. We also tested the scenario where the partial volume effects were not considered during the segmentation and still observed a consistent improvement of correlation between age and GM volume ratio in the harmonized images, and the details can be found in supplementary Fig. S3. More segmentation examples can be found in supplementary Fig. S4.

### 4.7. Harmonization improves consistency of PVS count ratio across MRI scanners on ADNI1

**Fig. 8.**
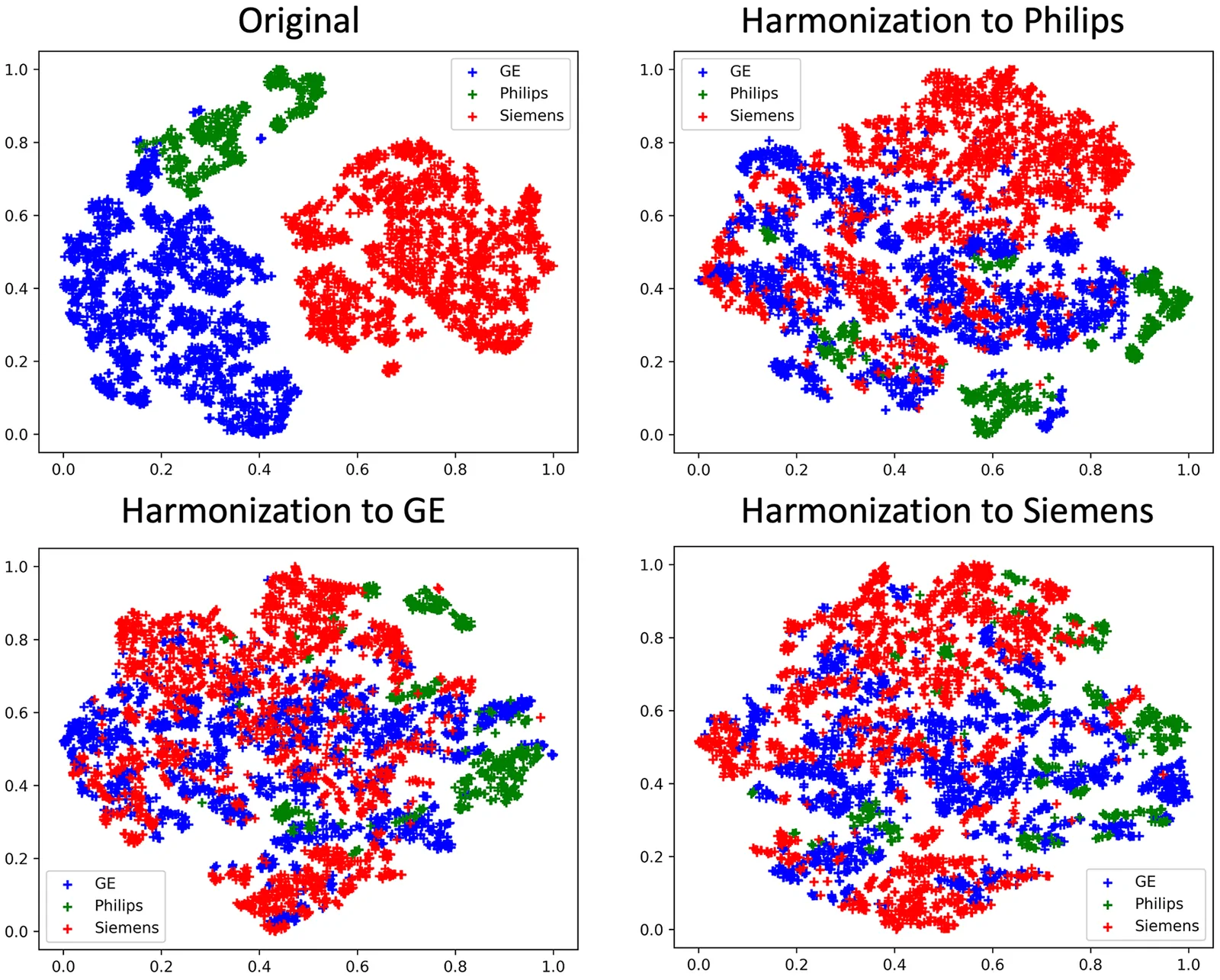
t-SNE visualization of harmonization effect on ADNI 1. In the original image space, there are identifiable three separated clusters of the domain embedding features visualized by t-SNE plot as shown in the top left corner. After the images get harmonized to either GE, Philips, or Siemens image domain, the separations disappear and all test data belong to one big cluster, which indicates the effectiveness of scanner effect harmonization. t-SNE was calculated on the testing data.

We used trained domain classifier to extract the domain embeddings from the images for the t-SNE calculation. As shown in Fig. 8, t-SNE visualizes the extracted domain embedding vectors of test data in the two-dimensional space for ADNI 1 dataset. Test images in the original image space have formed three distinct clusters as shown in the top left corner of Fig. 8. After the images get harmonized to either GE, Philips, or Siemens scanner effects, the separations disappear and all test data belong to one big cluster, which indicates that the harmonized images only contain one of the three scanner effects. Compared with multicenter image data of ABIDE II presenting diverse imaging features due to the different acquisition parameters, ADNI I dataset was acquired with the same acquisition protocol across different scanners which reduced imaging inhomogeneity at the acquisition level. Because the imaging features difference in the ADNI I dataset across different scanners are subtle, we used the domain embedding features extracted from a classifier for the t-SNE visualization instead of using the radiomics features.

**Fig. 9.**
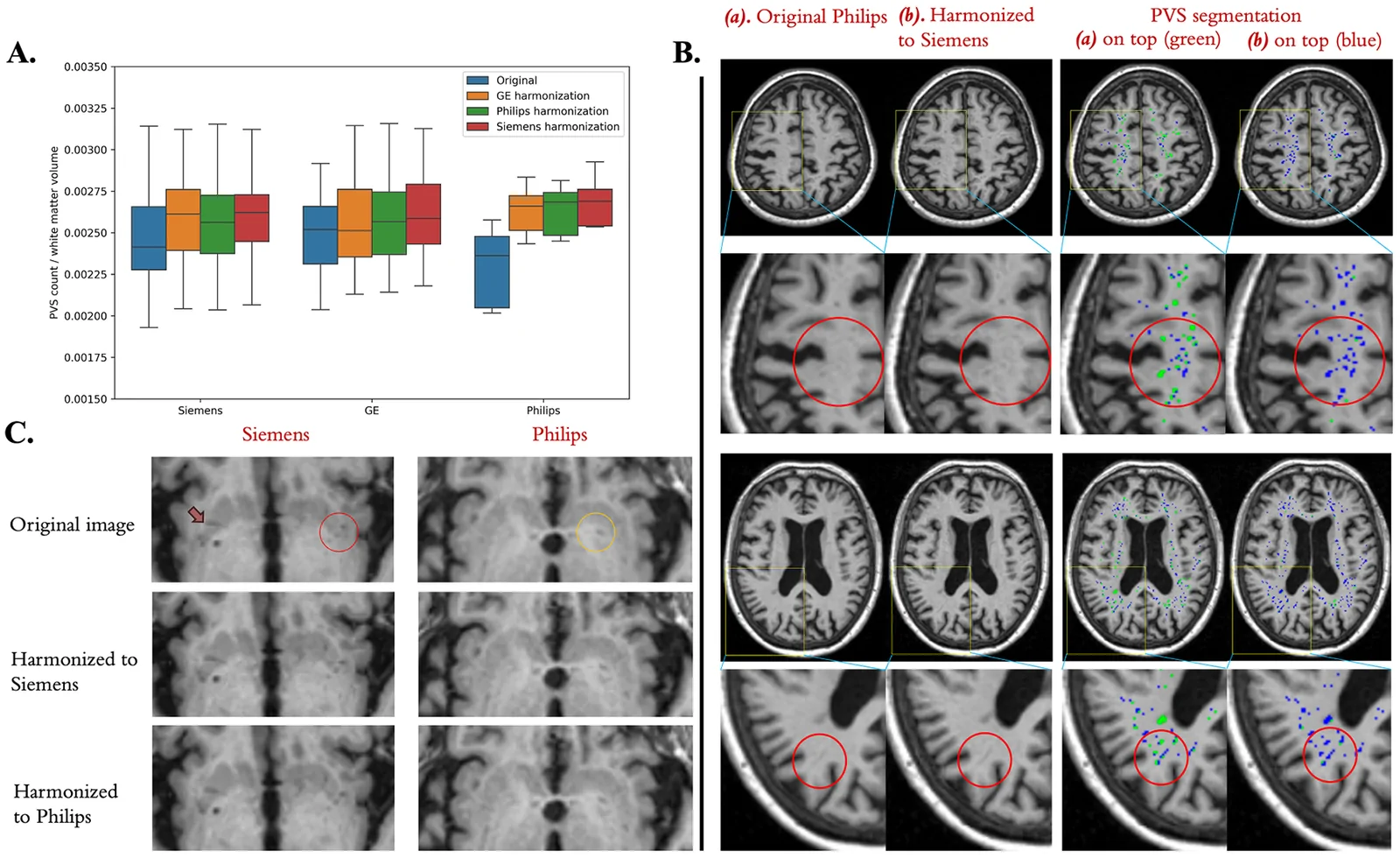
PVS segmentation quantification. A. Y-axis represents the PVS count ratio and X-axis represents the original scanner utilized to acquire the images. To generate the PVS count, the identified tubular structure regions by Frangi filter with less than 3 connected voxels were removed to remove the noise signals captured by Frangi filter. Harmonized images show reduced heterogeneity regarding PVS count ratio across scanners as show in orange, green and red boxplots compared to the blue boxplots. B. Two subjects’ axial slices were shown in this Fig.. Red circles indicate regions with visible increased PVS contrast. The last two columns show the PVS segmentation overlays in blue (harmonized image) and green (original image). Visible blue voxels (second to last column) indicate the PVS which were missed by the segmentation from the original image, qualitatively showing the harmonization effectiveness alongside with Fig. A. The same threshold was applied to both segmentations. C. Visual comparison between samples from Siemens and Philips and harmonized images respectively. Philips scanner produced more smoothed image with less sharp PVS boundaries comparing with the Siemens sample (red and yellow indicators). Such boundary sharpness was increased by harmonizing to Siemens and reduced by harmonizing to Philips.

As shown in Fig. 9 A., we observed inconsistent Frangi filter segmented PVS count/white matter volume distributions among images in ADNI 1, with lower PVS count/white matter volume ratio in the images acquired by Philips compared to images acquired from other two types of scanners. After the harmonization, PVS count ratio distribution became consistent across images acquired from all three scanners. Fig. 9 B. shows the original image acquired at Philips scanner, the image harmonized to Siemens scanner style and PVS segmentation overlays. The red circles indicate the region with increased sharpness of PVS boundaries in harmonized images compared to original images. As shown in the second to last column of Fig. 9 B, harmonized images produced more PVS signals (blue) which were not captured by Frangi filter on the original images. Since the harmonized images didn’t introduce significant false negative PVS signals compared with the original images (green signals in the last column), it further demonstrates that the additionally captured PVS signals are not just random noise which could be also observed in the Fig. 9A. with an aligned PVS distribution after the harmonization. The same Frangi filter threshold was applied to both segmentations.

The difference of the PVS boundary sharpness could be also observed in Fig. 9 C. which visually demonstrates more smoothed PVS boundaries of Philips sample compared with Siemens sample and increased PVS boundaries sharpness by harmonizing the image to Siemens style (red and yellow indicators).

### 4.8. Ablation study (learned condition improves the generated image quality)

**Fig. 10.**
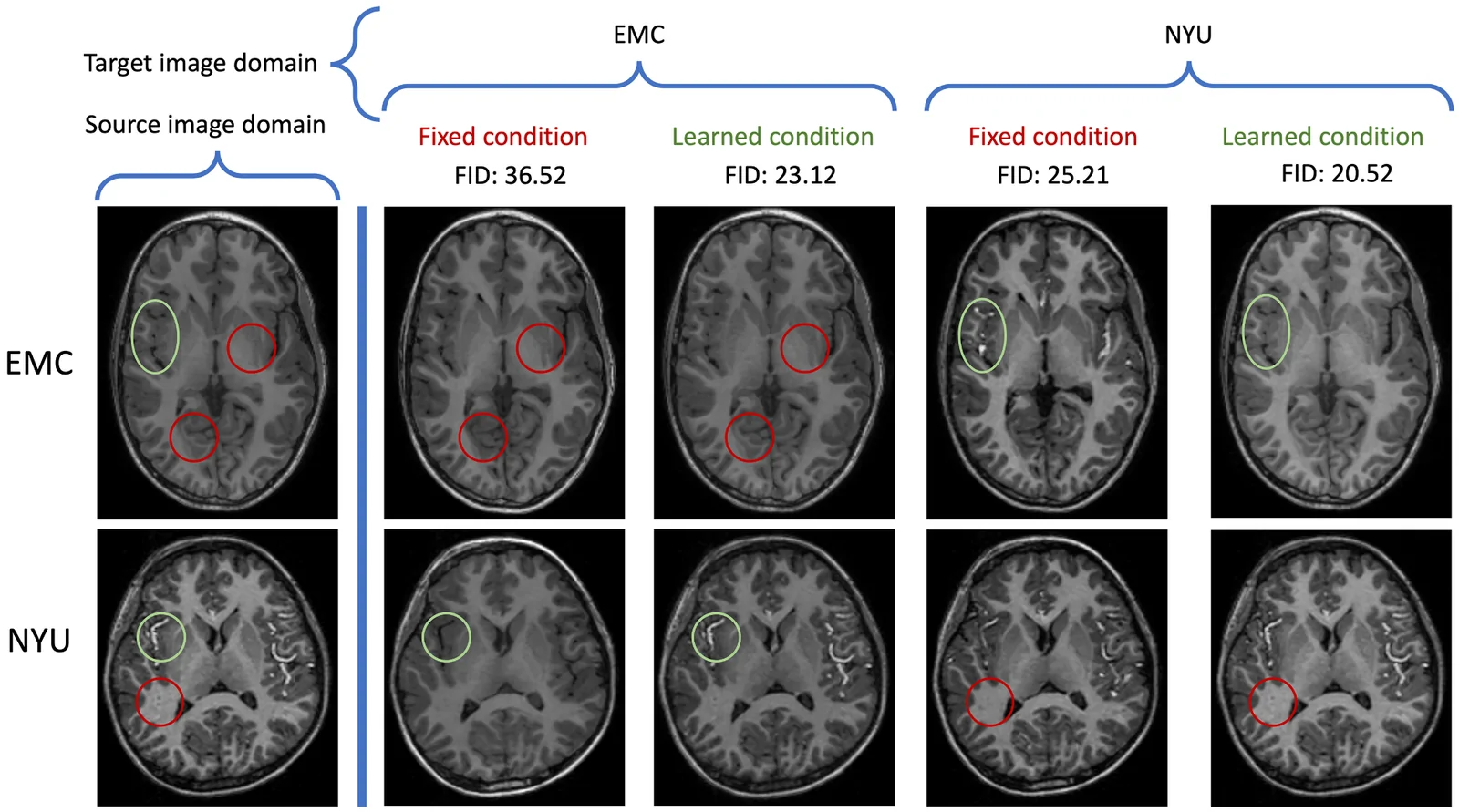
Qualitative comparison of harmonization results with fixed condition and learned condition. Green circles indicate that the artery signals got removed by harmonizing NYU → EMC data domain and generated by harmonizing EMC → NYU data domain with fixed condition (edge map), while learned condition reinforced consistent artery signals between the source image and the harmonized target image. Red circles indicate the areas with inferior performance of the preservation of anatomical details by conditioning on the fixed condition compared to the learned condition.

We study alternative denoising network conditioning by removing the condition extractor network and instead using the edge map as the condition *Y*. As shown in Fig. 10, samples from two image domains were presented and ‘Fixed condition’ represents the edge map conditioning. The edge map conditioning resulted in inconsistent anatomical details after harmonization, as evidenced by the removal of artery signals when harmonizing NYU data to the EMC data domain, and the appearance of artery signals when harmonizing EMC data to the NYU data domain, as indicated by the green circles. Also, the anatomical details were not fully preserved after the harmonization with edge map conditioning as indicated by red circles. In Table 2 we also reported inferior FID score of the proposed method with fixed condition compared to the learned condition.

## 5. Discussion

In this work we proposed a diffusion-based multi-domain harmonization framework which learns multiple diffusion trajectories correlated to image domains respectively. Diffusion model was conditioned on the learned domain invariant condition which guarantees the anatomical details unchanged during the harmonization. A skip sampling was introduced to shorten the sampling process. We quantitatively evaluated our proposed method on ABIDE II dataset by FID score for site effects harmonization and qualitatively demonstrated the superior performance of our method compared to two baseline GAN based models. Further, we evaluated our method on ADNI 1 dataset for scanner effects harmonization with an additional PVS segmentation downstream analysis to show improved consistency of PVS count ratio after the harmonization.

Given the recent advancements in diffusion-based models for high-fidelity image generation and multi-trajectory learning (Biroli et al., 2024), we aimed to investigate their potential application in neuroimage harmonization. Specifically, our aim was to address the challenges still faced by GAN-based methods, including multi-domain harmonization and artifact generation. The proposed method comprises a domain-invariant condition extractor ***C*** and a denoising network ***D_t_***. Both modules are governed by a domain embedding that defines the trajectory of the diffusion process. The extractor ***C*** was trained to extract the most domain invariant feature condition which contains anatomical information shared across batches and robust to the batch effects. These extracted domain-invariant features serve as the conditioning input for the denoising network ***D_t_*** , enabling it to generate images in the target domain’s imaging style through the controlled target diffusion trajectory. The denoising network ***D_t_*** acts as a powerful generative model, learning the domain-specific distribution, and plays a central role in the proposed harmonization framework. A visual representation of the diffusion process, conceptualized as the harmonization process, is provided in Fig. 2.

To further mitigate the influence of residual domain-specific features in the learned feature condition, we introduced an unlearning process. The unlearning process is framed as an adversarial learning paradigm, where the domain-invariant feature extractor ***C*** aiMS to generate features that are as domain-invariant as possible. These features are then evaluated by a discriminator *disc*. The resulting Nash equilibrium between ***C*** and *disc* signifies a balance in the adversarial training process, ensuring that the extracted features are effectively domain-invariant. The in-between-slice discrepancies in harmonized 3D data have been recently recognized as a limitation of 2D harmonization methods. To address this challenge, which adversely affects the image quality of the harmonized outputs, we introduced an additional 3D fusion module. This module is designed to integrate harmonized 2D slices into a coherent 3D representation, thereby mitigating potential inter-slice errors as shown in Fig. 6 C.

To assess the effectiveness of the proposed method, we conducted evaluations on two publicly available datasets: ADNI 1 and ABIDE II. For the ABIDE II dataset, we focused on a 4-site harmonization task. The results were analyzed both qualitatively and quantitatively and compared against two GAN-based baseline models. We demonstrated the superiority of the diffusion-based approach in multi-domain harmonization, as evidenced by the FID score, as well as in high-fidelity image harmonization, which was qualitatively validated by the absence of artifacts. For the ADNI 1 dataset, a 3-scanner harmonization task was evaluated, with the harmonization effect visualized using t-SNE plots of the latent embeddings of the domain classifier, as shown in Fig. 8.

Two downstream analyses were performed to evaluate the effectiveness of the proposed harmonization method: PVS segmentation on the ADNI 1 dataset and radiomics analysis on the ABIDE II dataset. In the ADNI 1 dataset, the distribution of the PVS count to white matter volume ratios exhibited inconsistencies across images, with those acquired using the Philips scanner showing a lower PVS count to white matter volume ratio compared to images obtained from the other two scanner types, as shown in 9A. PVS segmentation analysis also showed that, even though ADNI 1 dataset followed the same acquisition protocols with imaging inhomogeneity reduced, Philips scanner produced more smoothed images which reduces the PVS boundary sharpness compared with other two scanners. The reduced boundary sharpness causes the thresholding-based filtering method Frangi filter to segment less PVS signals from Philips scanner produced images and this inconsistency regarding the PVS count to white matter volume ratio was able to be alleviated through our proposed method.

In the radiomics analysis on the ABIDE II dataset, distinct clusters of radiomic features were clearly identifiable in the original image space for both WM and GM, as visualized using t-SNE, shown in Fig. 5. However, following harmonization, these clusters were no longer observed, suggesting that the harmonization process successfully reduced domain-specific variations between the different imaging sites. To quantitatively assess this improvement, we calculated voxel intensity density plots before and after harmonization, combined with Wasserstein Distance (WD) to evaluate the shift in voxel intensity distributions. The results demonstrated a more aligned voxel intensity distribution post-harmonization, further confirming the efficacy of the harmonization process in reducing inter-domain discrepancies and improving the consistency of imaging data across scanners.

Apart from GAN based methods including two baseline methods in this study and recent GAN based multi-domain harmonization developments (Cackowski et al., 2023; Liu et al., 2023; Roca et al., 2023b), our proposed method leverages the multi-trajectory learning and high-fidelity image generation of the diffusion model. We designed an explicit content-style separation by conditioning the controlled diffusion process on domain invariant features which are shared across multi-domain dataset and extracted using domain unlearning adversarial training along with the edge reinforcement. By conditioning the diffusion generative process on the domain invariant feature, we found the model is robust to overcome the artifacts generation which is evident in two GAN based baseline models. Even for the recent development of multi-center GAN based approach from (Roca et al., 2023b), the artifact signal generation at the prefrontal lobe of T1w images could be observed (second row and last column of Fig. 3. Roca et al), which aligns with our observation of CycleGAN model on our experimental dataset (Fig. 4). Artifacts generation would undoubtfully harm the image integrity of the harmonized images, as well as posing challenges for the data usability of such images towards additional downstream training such as super-resolution and segmentation tasks or even the radiologist’s diagnosis. Several causes could lead to the artifact generation including model collapse, using cycle consistency loss without explicitly defining the separation between anatomical content and domain-specific imaging feature biases. In this study, we demonstrated that using domain invariant conditioned diffusion model could overcome this issue. For a fair comparison, we compared our method with the original CycleGAN model adapted to MRI image processing and the Seg-Renorm model which incorporates a brain ROI segmentation module to improve the generated image quality. Even though Seg-Renorm generated images have significantly reduced artifacts, we still observed the universal presence of the strap patterns which are not anatomically consistent. Overall, the proposed method has the best FID score comparing to the baseline models. Our ablation study also demonstrated the superior anatomical consistency in the harmonized images by conditioning on the learned domain invariant condition compared with fixed edge map condition.

Our volumetric analysis focusing on the correlation between age and GM volume ratio showed that the proposed harmonization method warrants the anatomical consistency while correcting the domain biases to improve the correlation between age and GM volume ratio. Similar observation of the strengthened negative correlation between age and GM volume ratio for the effective harmonization was also reported in Fig. 5. Roca et al, while the histogram matching based method showed reduced linear relationship. While Roca at al analyzed the generalization dataset with the age range between 19 – 80 years old, we focused on the dataset covering age range between 5 – 35 years old. The volumetric analysis also revealed the segmentation error which produced GM volume ratio outliers in NYU samples and such segmentation error was able to be fixed by harmonizing the NYU data to other three reference sites. One of the potential causes of the segmentation error of the automatic pipeline is the remaining intensity inhomogeneity (bias field) in the images. The bias field introduces smooth, low-frequency variations in intensity across the MRI image, which can distort voxel intensities and affect tissue segmentation accuracy. Bias field correction is generally included as a preprocessing step of the automatic neuroimaging pipeline, however, intensity inhomogeneity could still partially remain after this preprocessing step which results in the errors in brain extract or segmentation. Careful selection of the reference domain and quality control should also be considered for the harmonization task.

## 6. Limitations

ADNI 1 dataset used for the experiment was acquired under the 1.5T field strength with three imaging vendors; ABIDE II used for the experiment was acquired under 3T field strength from four imaging sites. For our experiment, the learned domain condition edge map for the diffusion model has been only tested for the images acquired under the same field strength and harmonization focused on the radiomic texture heterogeneities. In this study, we did not perform the harmonization tasks on images acquired under different field strengths by incorporating the super-resolution, nor testing the reliability of learned domain condition edge map on such dataset. The proposed method was only tested on the T1w images, however, the theory should be held for other structural neuroimaging modalities such as T2-weights MRI and Fluid-attenuated inversion recovery (FLAIR) MRI images. We will be looking forward to testing the proposed method on more broad range of imaging modalities and imaging datasets.

Because there was no traveling subject involved for this study, we were not able to use the paired voxel-level evaluation metrics like peak signal-to-noise ratio (PSNR), structural similarity (SSIM), or root-mean-squared-error (RMSE) to quantify the harmonization results. We also didn’t test the proposed method in the scenario where one of the imaging sites includes severe anatomical anomalies such as the presence of certain tumor which has unique MRI imaging textures compared with healthy tissues. For this study, we only tested the harmonization performance in-between the healthy subjects and ASD subjects. However, the prudence should be warrantied for harmonizing the oncology data with healthy imaging data, or with different type of pathological imaging data which has well known imaging texture or anatomical biases, and rigorous evaluation should be performed for such harmonization tasks to guarantee the anatomical consistency and harmonization accuracy.

## 7. Conclusion

In conclusion, our study demonstrates the efficacy of using a diffusion model to address the multi-domain neuroimaging harmonization challenge while preserving anatomical and biological details. The conditional diffusion generative process enables the proposed method to harmonize multi-domain imaging datasets, with domain embeddings controlling the harmonization within a single unified model. Our approach specifically targets the harmonization of imaging texture heterogeneity caused by technical variability across large, multi-center datasets. Such datasets are crucial for investigating the relationship between clinical pathologies and differentiating their long-term impacts on the clinical manifestations of diseases. We performed both quantitative and qualitative evaluations of the harmonization effects and anatomical consistency, including volumetric analysis and PVS segmentation analysis, using two publicly available datasets: ABIDE II and ADNI 1. Additionally, the diffusion model exhibited robustness in mitigating artifact generation by leveraging domain-invariant conditioning and domain-variant adaptation, thereby ensuring the reliability and usability of the imaging harmonization result

## Supplementary Material

1

Supplementary material associated with this article can be found, in the online version, at doi:10.1016/j.neuroimage.2025.121297.

## Figures and Tables

**Table 1:** Acquisition parameters and demographic information of selected sites from ABIDE II.

	EMC	ETH	GU	NYU
Field Strength	3T	3T	3T	3T
Manufacture	GE	Philips	Siemens	Siemens
Model	MR750	Achieva	TriTim	Allegra
Headcoil	8ch	32ch	12ch	8ch
Sequence	IR-FSPGR	3D TFE	MPRAGE	3D TFL
Slice Thickness [mm]	0.9	0.9	1	1.33
Slice In-Place Resolution [mm2]	0.9 × 0.9	0.9 × 0.9	1 × 1	1.3 × 1.0
Age (years old)	6 - 11	14-31	8.1-13.9	5.2-34.8
Sample size	48	35	91	78
Autism Spectrum Disorder (ASD)	24	13	45	40
Control	24	22	46	38
Sex (male)	38	35	71	71
Sex (female)	10	0	20	7

**Table 2:** Quantitative comparison with baseline models and the ablation model. The Bold font indicates the best Fréchet inception distance (FID) score and underline indicates the second best. The qualitative comparison can be found in Fig. 4 and Fig. 8.

FID ↓ Method	Others→EMC	Others→ETH	Others→GU	Others→NYU
Source, Target	39.6	56.2	36.7	37.6
CycleGAN (baseline)	**23.1**	40.1	18.45	31.1
Seg-Renorm (baseline)	25.9	39.4	15.2	28.9
Ours (Edge map)	36.5	46.8	30.6	25.2
**Ours (Learned condition)**	**23.1**	**38.9**	**12.1**	**20.5**

## Data Availability

Data will be made available on request.
