# Supplementary material for: Diffusion based multi-domain neuroimaging harmonization method with preservation of anatomical details
Source: Neuroimage. Author manuscript; Available in PMC 2026 Aug 24. (PMC13500686; doi:10.1016/j.neuroimage.2025.121297)
Supplement: Supplementary file 1 [file NIHMS2176773-supplement-1.docx]

# Supplementary material

Figure S1. Architecture of the fusion module. The fusion module is a 3D residual convolutional neural network with the skip connections between early and late CNN layers. The fusion module doesn’t have down-sampling layers, which was designed to maintain imaging details while reducing the in-between-slice difference as originally introduced by (Zuo et al., 2021). The input of the fusion module is the stacked harmonized images through axial, coronal and sagittal slices, which makes the input image size is $256\times256\times256\times3$. The fusion module was trained to fuse the stacked 2D harmonized images with 3 channels into a coherent singe channel 3D image without in-between-slice difference to optimize the L1 loss between the output and the reference image sample. Because the fusion module was trained using the samples of harmonization task where the source image and reference image are the same, the training process of fusion module is the supervised training.

Figure S2. Visual example of the training sample and inference results of the fusion module. The first row shows one sample for the fusion module training where the last three images are the inputs of fusion module with visible in-between-slice difference (harmonized image through axial, coronal, and sagittal slices respectively), the first image is the target which is the actually image sample from site 2, and the second image is the output which is the fused harmonization results (red box indicates the image that should be ideally identical as the site 2 sample). The rest of the rows show inference results with site 2 sample being harmonized to the target sites respectively. Note that the proposed method was trained using axial slices only and used for coronal and sagittal slices harmonization directly.

Figure S3. Volumetric analysis between the GM volume ratio and age for ABIDE dataset. A. the segmentation results from the tissue segmentation pipeline which doesn’t consider the partial volume effects. B. the segmentation results with the consideration and correction of partial volume effects. Both plots indicate the harmonization increased the linear relationship between the GM volume ratio and age regardless of the accuracy of the segmentation results.

Figure S4. Visual examples of the brain tissue segmentation from original image and harmonized images respectively (top example), and segmentation outlier existing in NYU site (bottom example).
